# Revolutionizing the Management of Large-Core Ischaemic Strokes: Decoding the Success of Endovascular Therapy in the Recent Stroke Trials

**DOI:** 10.3390/jcdd10120499

**Published:** 2023-12-18

**Authors:** Gareth Zigui Lim, Jonathan Yexian Lai, Christopher Ying Hao Seet, Carol Huilian Tham, Narayanaswamy Venketasubramanian, Benjamin Yong Qiang Tan, Mingxue Jing, Joshua Yee Peng Yeo, May Zin Myint, Ching-Hui Sia, Hock Luen Teoh, Vijay Kumar Sharma, Bernard Poon Lap Chan, Cunli Yang, Andrew Makmur, Shao Jin Ong, Leonard Leong Litt Yeo

**Affiliations:** 1 Department of Neurology, National Neuroscience Institute, Tan Tock Seng Hospital, Singapore 308433, Singapore; 2 Raffles Neuroscience Centre, Raffles Hospital, Singapore 188770, Singapore; ramani_nv@rafflesmedical.com; 3 Division of Neurology, Department of Medicine, National University Health System, Singapore 119228, Singaporevijay_kumar_sharma@nuhs.edu.sg (V.K.S.); bernard_chan@nuhs.edu.sg (B.P.L.C.);; 4 Yong Loo Lin School of Medicine, National University of Singapore, Singapore 119077, Singapore; 5 Department of Cardiology, National University Heart Center, Singapore 119228, Singapore; 6 Department of Diagnostic Imaging, National University Health System, Singapore 119228, Singapore

**Keywords:** ischaemic stroke, endovascular treatment, radiology, CT, MRI, acute, large infarcts

## Abstract

Endovascular therapy (EVT) has revolutionized the management of acute ischaemic strokes with large vessel occlusion, with emerging evidence suggesting its benefit also in large infarct core volume strokes. In the last two years, four randomised controlled trials have been published on this topic—RESCUE-Japan LIMIT, ANGEL-ASPECT, SELECT2 and TENSION, with overall results showing that EVT improves functional and neurological outcomes compared to medical management alone. This review aims to summarise the recent evidence presented by these four trials and highlight some of the limitations in our current understanding of this topic.

## 1. Defining the Role of EVT in Large Infarct Core Strokes

Since 2015, endovascular therapy (EVT) has continued to revolutionize the management of acute ischaemic strokes with large vessel occlusion [1,2,3,4,5,6,7,8]. In stroke patients who fulfil strict clinical and radiological criteria, it has been consistently effective at improving functional outcomes, which reduces disability and length of hospital stay. Various international and local clinical consensus guidelines have established clear recommendations for performing EVT in patients that either present (A) within a 6-h window from stroke onset with an Alberta stroke programme early CT score (ASPECTS) of ≥6 on non-contrast CT (NCCT) or (B) within a 6 to 24 h time window with clinical- core-penumbra mismatch that fulfils clinical perfusion criteria established by the landmark DAWN (DWI or CTP Assessment with Clinical Mismatch in the Triage of Wake-up and Late Presenting Strokes Undergoing Neurointervention with Trevo) and DEFUSE-3 (The Endovascular Therapy Following Imaging Evaluation for Ischaemic Stroke) trials [9,10].

**Figure 1:**
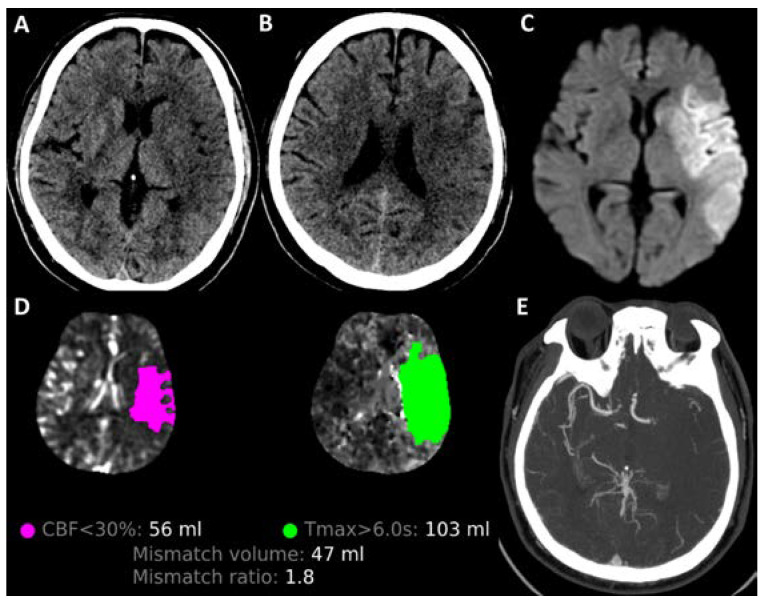
A large infarct core volume (LICV) stroke is demonstrated in this figure. There is a left middle cerebral artery territory (MCA) infarct with loss of grey–white differentiation in the left caudate, internal capsule, insula, M2 (**A**), as well as left M5 and M6 regions (**B**), giving an ASPECTS of 4. MRI brain shows corresponding DWI hyperintensities in the left MCA territory (**C**). CT perfusion imaging processed by RAPID software (v 1.3.3) shows the ischaemic core and perfusion deficit. The ischaemic core (pink) is 56 mL while the tissue at risk of infarction (green) is 103 mL, giving a large mismatch ratio of 1.8 (**D**). CT vessel angiogram shows a left M1 large vessel occlusion (**E**).

Since then, many have challenged the conventional limits of EVT by time and tissue windows, with emerging evidence suggesting its benefit in large infarct core volume (LICV) strokes. LICV strokes, defined as strokes with ASPECTS < 6 or infarct core volume ≥ 50 mL (Figure 1), account for more than 25% of ischaemic strokes that present at hospitals [11]. Its management has traditionally only been supportive, with many of the early stroke trials excluding LICV strokes based on the assumption that these patients already have large irreversible infarcted tissue and that EVT is associated with higher mortality and bleeding risks. However, more recent pooled meta-analyses, observational small studies [12], including those conducted by the HERMES (Highly Effective Reperfusion Evaluated in Multiple Endovascular Stroke Trials) collaboration have highlighted the potential benefit for EVT in LICV strokes. This offers a glimpse of hope for the management of these patients, who would otherwise face devastating irreversible outcomes.

Of patients with ASPECTS < 6, previous studies have suggested that EVT may have the largest benefit in those with ASPECTS 3–5 and be futile in those with ASPECTS 0–2 [13,14]. In patients with ASPECTS 0–2, meta-analyses have shown that medical management alone led to better outcomes than EVT. However, these studies had small sample sizes (less than 50 patients) and were not sufficiently powered to examine LICV stroke patients, making it difficult to draw definitive conclusions. Patients with ASPECTS 0–2 constitute a substantial proportion of almost 10% of all strokes; therefore additional studies are needed to clarify the role of advanced imaging in determining those who may potentially benefit from EVT.

## 2. Imaging Considerations in Large Infarct Core Strokes

Many believe that the unexpected benefit we are now seeing with EVT in LICV strokes is largely due to the limitations of our current imaging’s ability to differentiate irreversibly infarcted core from salvageable penumbra. The ASPECTS scoring tool, traditionally used on NCCT to assess infarct volume may not be as accurate as we thought. Some studies have shown variations in its inter-rater agreement depending on the reader’s experience, background and imaging settings [15,16]. This may erroneously classify patients with smaller strokes as LICV strokes and vice versa. This is in contrast to diffusion-weighted imaging (DWI) on MRI which has substantially better inter-rater homogeneity and accuracy at scoring ASPECTS—MRI-ASPECTS have been reported to score 1 point lower than CT-based ASPECTS scoring [17,18]. Understandably, obtaining an MRI is time consuming and not readily accessible in most hospitals worldwide. Perhaps one way around this is to utilise automated software programs such as Frontier, Brainomix and RAPID ASPECTS that employ artificial intelligence and deep learning to reduce the variability of ASPECTS evaluation [19,20]. These programs have since been adopted in many hospitals’ stroke management protocols.

Relying on NCCT alone to predict treatment response is inadequate, with studies showing that low ASPECTS on NCCT does not necessarily guarantee larger core or poor functional outcomes after intervention. Broocks et al. showed that the degree of tissue net water uptake (NWU) on NCCT—a variable not usually measured on routine binary ASPECTS scoring—is a quantitative biomarker of ischaemic oedema and can be used to predict functional outcomes in LICV strokes [21]. Different patients with LICV strokes can have different degrees of NWU for the exact same ASPECTS. Lower tissue attenuation (low NWU) indicates lesser ischaemic oedema and is associated with better functional outcomes with recanalization. On the other hand, higher NWU is associated with malignant oedema and poorer outcomes [22]. If NWU was measured, we could further stratify a subgroup of patients with low ASPECTS LICV strokes that may benefit more from EVT.

CT perfusion (CTP) imaging, touted as the breakthrough tool for tissue-based decision making in acute stroke management, also has its limitations. Despite the extensive evaluation carried out to determine optimal threshold maps for core and penumbra assessment, proposed definitions are still non-conclusive, partly due to study heterogeneity and the variety of software used [23]. The accuracy of CTP on core estimation has also been challenged, with studies showing that it can over-estimate infarct core volume in strokes that present early from symptom onset—a concept known as the ghost core [24]. Poor collateral supply in areas with hypoperfusion could be wrongly considered as already infarcted on CTP. Moreover, CTP provides a snapshot of the haemodynamic state which is in constant flux and can only be used as a surrogate for truly infarcted tissue [25]. Lastly, several studies have demonstrated the reversibility of DWI, showing that DWI lesions, once thought to indicate irreversible cytotoxic oedema from infarcted tissue, can potentially be reversed after prompt recanalization and reperfusion. This is estimated to be present in as many as 1 in 4 patients, with complete reperfusion and shorter imaging time to recanalization being independently associated factors [26]. The good response of LICV strokes to EVT could conceivably be explained in part by the concept of DWI reversibility.

These concepts have exposed the limitations in our current understanding of what constitutes irreversible infarct and salvageable penumbra. Have these misconceptions deprived many LICV stroke patients of the treatment they needed all along?

## 3. HERMES Pooled Data and Meta-Analysis Preceding the RCTs

Since as early as 2016, data from pooled meta-analyses, observational cohort studies and small randomised controlled trials (RCT) have hinted at the benefit of EVT in LICV strokes. The THRACE trial was the earliest RCT to include patients with LICV; it randomised patients regardless of ASPECTS score to receive either EVT and alteplase or alteplase alone. It included more than 13% of patients with ASPECTS < 6 and showed that intravenous alteplase combined with EVT improved functional outcomes in these patients [7]. A subgroup analysis of this study also showed that the majority of patients with DWI volumes of >70 mL had favourable outcomes with EVT [27]. In a meta-analysis of 7 RCTs, the HERMES group showed that EVT achieved better outcomes at 90 days than medical therapy alone across a broad range of baseline imaging categories including ASPECTS 3–6 [13] or when CT perfusion or DWI core volumes were ≥70 mL [28]. These results were echoed by other meta-analyses: one analysed 17 studies and 1378 patients (1194 of which underwent EVT) with ASPECTS 0–5 and showed that EVT was associated with higher rates of patients achieving modified Rankin scale (mRS) 0–2 [29]; another by Sarraj et al. analysed 12 studies and demonstrated increased functional independence rates with EVT for patients with ASPECTS < 6 or ischaemic core > 50 mL [30]. Despite encouraging results, the data from these analyses tended to be too heterogenous with small sample sizes, hence the call for larger and more focussed RCTs to explore this further.

## 4. RESCUE-Japan LIMIT RCT

**Figure 2:**
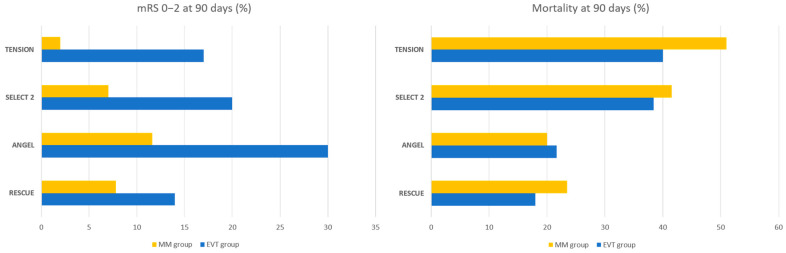
mRS 0–2 at 90 days and mortality at 90 days across the trials.

A summary of the key points from the recent five large RCTs is shown in Table 1 and Figure 2. In mid-2022, the RESCUE-Japan LIMIT RCT (Recovery by Endovascular Salvage for Cerebral Ultra-acute Embolism–Japan Large Ischaemic Core Trial) [31] was the first of these that showed the benefits of EVT in LICV Japanese stroke patients compared to medical management alone. This study recruited a total of 203 patients presenting within 24 h of the last known well, with a NCCT or DWI-MRI ASPECTS of 3–5. If patients presented within 6 to 24 h, there had to be no corresponding MRI FLAIR hyperintensity. The median ASPECTS was 3 and the median NIHSS score was 22. Most patients (more than 60%) presented within 4.5 h and most (about 86%) were selected based on MRI. Intravenous alteplase at 0.6 mg/kg was administered when indicated as per local clinical guidelines. The results showed that EVT achieved a higher rate of mRS 0 to 3 at 90 days (31% vs. 12.7%, odds ratio (OR), 2.43), with a shift of mRS ordinal categories also favouring the EVT arm (OR, 2.42). However, the rates of symptomatic intracerebral haemorrhage (ICH) within 48 h and any ICH within 48 h were almost double in the EVT compared to the medical management arm, albeit only the latter being statistically significant (58% vs. 31.4%, relative risk (RR), 1.85). The results from this study were promising; although the frequent use of MRI in RESCUE-Japan LIMIT where MRI-ASPECTS tended to over-estimate infarct volume, the lack of perfusion imaging and lower thrombolysis dose were minor limitations that made it less generalisable to other countries.

## 5. ANGEL-ASPECT RCT

ANGEL-ASPECT (Endovascular Therapy in Acute Anterior Circulation Large Vessel Occlusive Patients with a Large Infarct Core) [32], conducted in China, was the second RCT published on this topic that echoed the outcomes of the RESCUE-Japan LIMIT trial. A total of 456 patients presenting within 24 h of the last known well were enrolled in this study. These patients had either a NCCT ASPECTS of 3–5, with an infarct core volume (cerebral blood flow (CBF) < 30%) of at least 70–100 mL if their ASPECTS was either <3 or >5. The median ASPECTS was 3, the median infarct core size was about 60 mL and the median NIHSS score was 16. About 63% of patients presented in the late window (6 to 24 h) and 28% received intravenous thrombolysis when indicated, as per local clinical guidelines. The shift in mRS distribution at 90 days favoured the EVT group (OR 1.37), with a larger proportion of patients achieving an mRS of 0 to 2 (47% vs. 33%, OR 1.50). Similar to RESCUE-Japan LIMIT, the rates of symptomatic ICH or any ICH within 48 h were higher in the EVT group (OR 2.07 and 2.71, respectively), with only the latter being statistically significant. The two RCTs conducted in the Asian population yielded surprisingly similar results, hinting at the benefit of EVT in LICV strokes but with caution advised, given the slightly higher bleeding rates.

## 6. SELECT2 RCT

SELECT2 (Randomised Controlled Trial to Optimise Patient’s Selection for Endovascular Treatment in Acute Ischaemic Stroke) [33] was the first RCT that looked at the benefit of EVT in LICV strokes in the Western population. It recruited a total of 352 predominantly white patients from hospitals in the United States, Canada and Europe. All patients presented within 24 h of last known well and had both a NCCT and perfusion study. Imaging inclusion criteria were the presence of either unfavourable ASPECTS (ASPECTS 3–5) or unfavourable perfusion (infarct core ≥ 50 mL). The median NIHSS score was 19; 78% of patients had a LICV stroke (ASPECTS ≤ 5 or infarct core ≥ 50 mL) and about 20% of patients received intravenous alteplase. Compared to medical management, the shift in mRS distribution at 90 days favoured the EVT group (OR 1.51) and a greater number of patients in the EVT group achieved functional independence (20% vs. 7%, RR 2.97). Subgroup analysis showed no heterogeneity of treatment effect across all groups, including in patients with very large perfusion ischemic cores of >100 mL or >150 mL, in those with large perfusion ischaemic cores coupled with low ASPECTS and even in those with small mismatch volumes. Around of 18% of patients in the EVT group had procedure-related complications, while symptomatic ICH at 24 h was low in both groups (0.6% vs. 1.1%, RR 0.49), affecting only 1 patient in the EVT group. The SELECT2 trial exposed the limitations of perfusion imaging at predicting irreversible infarct core [34], but also provided reassuring results that supported the use of EVT in LICV strokes in the Western population, with surprisingly lower rates of bleeding complications compared to RESCUE-Japan LIMIT and ANGEL-ASPECT. Although the lower bleeding complications were initially attributed to the low thrombolysis rate and better baseline ASPECTS scores in the SELECT2 population, the subsequent RCT did not have similar limitations.

## 7. TENSION RCT

The most recent of the EVT in LICV stroke trials—TENSION (The Efficacy and Safety of Thrombectomy in Stroke with extended lesion and extended time window) [35], was published in October 2023. It recruited a total of 253 patients predominantly from Europe and one site in Canada. All patients presented within 12 h of the last known well, had NIHSS scores of ≤26 and ASPECTS of 3–5. Extended imaging was not used in this trial; 82% of patients had NCCT scans only while 18% had MRI. The median NIHSS score was 18–19, median ASPECTS was 3–4 and about 36% of patients received intravenous alteplase. Once again, the shift in mRS distribution at 90 days favoured the EVT group (OR 2.58) with a larger proportion of patients achieving independent neurological outcomes (mRS ≤ 2) (17% vs. 2%, OR 7.16). Rates of symptomatic ICH were low, occurring in 5% of patients in both groups. Rates of any adverse safety events were significantly lower in the EVT group (55% vs. 70%). This was the first trial to show a statistically significant survival benefit in the EVT group, with death or dependency (mRS 4 to 6) at 90 days being lower in the EVT group (69% vs. 87%, OR 0.34). It was also the only RCT thus far to evaluate the effect of EVT on patient functional health status, quality of life and mental health assessed by self-reported questionnaires; it showed a statistically significant positive effect on patient health on most performance scales. In its subgroup analysis, it also showed a trend for the benefit for EVT in LICV strokes of ASPECTS 0–2, with a favourable point estimate of the OR (OR 1.51; Confidence Interval (CI) 0.44–5.19). Almost all the patients in TENSION had LICV strokes assessed on NCCT scans without the use of advanced imaging. This is crucial, as it supports the use of NCCT for decision-making in these patients—a result likely to be welcomed by most hospitals worldwide where advanced imaging is not readily accessible.

## 8. TESLA RCT

Preliminary results from a fifth RCT were available but yet to be published at the time of writing. The TESLA (Thrombectomy for Emergent Salvage of Large Anterior Circulation Ischaemic Stroke) trial [36], conducted in the United States, recruited a total of 300 patients presenting within 24 h of stroke onset that had NCCT ASPECTS of 2–5. Similar to TENSION, all patients underwent NCCT scans. Its preliminary results showed that patients treated with EVT did slightly better on a 90-day utility-weighted mRS score (2.93 in the EVT group vs. 2.27 in the MM group); however, the Bayesian prespecified probability of superiority was 0.957, which was below the level of >0.975 which was needed to declare efficacy. Some secondary outcomes such as the proportion of patients with an mRS of 0 to 3 at 90 days and the rate of major neurological improvement at day 5 to 7 significantly favoured the EVT group. Although there were no significant differences between 90-day mortality (35.3% vs. 33.3%) or symptomatic ICH (3.97% vs. 1.34%) between the two groups, the rates of parenchymal haematoma, haemorrhagic infarction and subarachnoid haemorrhage were significantly higher with intervention. Despite the negative primary endpoint in TESLA, the overall treatment effect weighs in favour of EVT, especially when the results from the preceding trials and subsequent meta-analyses [37,38] analysing data from RESCUE-Japan LIMIT, ANGEL-ASPECT, SELECT2 and TESLA are taken into consideration.

## 9. Summary and Discussion of Key Findings

The cumulative published results of RESCUE-Japan LIMIT, ANGEL-ASPECT, SELECT2 and TENSION have demonstrated that EVT in LICV strokes performed within 24 h of symptom onset is safe and associated with better neurological outcomes than medical management alone. These results were supported by two recent meta-analyses [37,38] which showed significantly better outcomes in support of EVT. Although the results of RESCUE-Japan LIMIT prompted early termination of the SELECT-2 and ANGEL-ASPECTS trials, both analyses demonstrated improvement in a 1-point mRS shift at 90 days as well as mRS 0–2 rates despite their limited sample sizes. Although not powered for subgroup analyses, all four trials showed similar benefits for EVT across all subgroups, including age, sex, time of last seen well to presentation, NIHSS score and whether or not intravenous thrombolysis was used. Results across the trials were also similarly in favour of EVT regardless of whether perfusion imaging was used or not. Between 19% to 36% of patients were administered intravenous thrombolysis in the RCTs. As stroke guidelines preclude the use of thrombolysis in low ASPECTS ischaemic strokes, it was difficult to specifically study the impact of thrombolysis in this patient cohort. Furthermore, slightly different definitions of symptomatic ICH were used by the included RCTs, making accurate comparisons of safety outcomes difficult. Nevertheless, the subsequent meta-analysis by Palaiodimou et al. [37] showed that the use of thrombolysis did not affect outcomes in the studies. The rates of successful reperfusion and thrombolysis in cerebral infarction grades (TICI 2b–3) were high, ranging from 81% to 86% in the trials. Although this was consistent with successful reperfusion rates in other thrombectomy studies, the impact of reperfusion status on outcomes was not analysed in detail in these trials. Additionally, blood pressure targets in the trials were guided by local practice and not uniform across institutions. The impact of blood pressure control on outcomes could have also been analysed in greater detail. Patient-directed quality-of-life measures would also be useful to help understand the true benefit of EVT in these patients; thus far, TENSION was the only trial to assess this.

Overall, the safety outcomes across the trials were acceptable. Slightly higher rates of ICH were seen in the Asian but not the Western patient population. The reason for this is uncertain, but previous studies have attributed a higher prevalence of intracranial atherosclerosis in Asians rendering the need for more EVT passes as the reason for greater EVT-associated bleeding complications [39]. Nevertheless, adverse outcomes were generally low and similar between both groups; the benefit of EVT, despite its potential risks in LICV strokes, still outweighs the all-too-familiar bleak outcomes of an untreated patient. Perhaps, as described by Ballout [34], the magnitude of the EVT treatment effect shown in these trials has simplified and democratized the EVT selection process—should EVT now be offered to all stroke patients with large vessel occlusion of the anterior circulation and ASPECTS > 2?

## 10. Concluding Remarks

Despite the positive results demonstrated by all four published RCTs, they each had slightly different methodologies and selection criteria—differing in ethnicity of the patient population, imaging modality (CT vs. MRI), time windows (early vs. late), and whether or not perfusion imaging was used [40]. This has implications for how we should interpret the minor differences between each of the trials’ results, which should be considered in the context of each trial’s imaging and clinical inclusion and exclusion criteria. Perhaps, there may well be a demographic or clinical correlate that has yet to be identified in this cohort of patients. 

Although meta-analyses of the recent trials showed slightly higher rates of symptomatic ICH in the EVT group, EVT still conferred a significantly greater clinical benefit than medical management alone. We await with anticipation the published data of the other LICV EVT trials, peer reviews, and subsequent pooled analyses, which may provide us with a more complete understanding of this topic. Nevertheless, the evidence thus far supports the need to change the way we manage patients with LICV stroke and also the need to update the existing stroke guidelines to reflect this evidence.

## Figures and Tables

**Table 1:** Summary of key findings in the recent large stroke EVT trials.

Trial	RESCUE-Japan LIMIT	ANGEL-ASPECTS	SELECT2	TENSION	TESLA (Unpublished)
Country(ies)	Japan	China	United States, Canada, Europe	Canada, Europe	United States
Size	101 EVT, 102 MM	231 EVT, 225 MM	178 EVT, 174 MM	125 EVT, 128 MM	152 EVT, 148 MM
NIHSS score	≥6	6–30	≥6	<26	≥6
Age	>18	18–80	18–85	>18	18–85
Imaging criteria	1. NCCT or DWI-MRI ASPECTS 3–5 175/203 had MRI (86%), rest had only NCCT Most had ASPECTS 3	1. NCCT ASPECTS 3–5 or 2. NCCT ASPECTS > 5 (>6 h) and infarct core 70–100 mL or 3. ASPECTS < 3 and infarct core 70–100 mL Most had CT and CTP, 38 had MRI Most had ASPECTS 3	1. NCCT ASPECTS ≥ 6 and infarct core ≥ 50 mL 2. NCCT ASPECTS 3–5 and infarct core ≥ 50 mL or 3. NCCT ASPECTS 3–5 and infarct core < 50 mL or 97% had CTP, rest had MRI Median ASPECTS 4	1. NCCT or DWI-MRI ASPECTS 3–5 82% had NCCT only, 18% had had MRI	1. NCCT ASPECTS 2–5
Thrombolysis	56/203 (27%) given alteplase	129/455 (28%) given alteplase (1 given urokinase)	67/351 (19%) given alteplase	93/253 (36%) given alteplase	Yes, unclear how many
Time window	Within 6 h from LKW or within 6 to 24 h from LKW (FLAIR-) Most presented within 4.5 h	Within 0 to 24 h from LKW Most presented within 6–12 h	Within 0 to 24 h from LKW Median time interval—9 h from LKW	Within 0 to 12 h from LKW Median time from symptom onset to groin puncture 4.2 h	Within 24 h of stroke onset
Key outcomes	mRS 0 to 3 at 90 days was higher in EVT group (31% vs. 12.7%; RR 2.43, CI 1.35 to 4.37) Shift of mRS ordinal categories favoured the EVT group (OR 2.42, CI 1.46 to 4.01)	mRS shift towards better outcomes at 90 days favoured EVT (OR 1.37, CI 1.11 to 1.69) 30% of EVT vs. 11.6% of MM had MRS 0 to 2 at 90 days (RR 2.62, CI 1.60 to 4.06)	mRS shift towards better outcomes at 90 days favoured EVT (OR 1.51, CI 1.20 to 1.89) 20% of EVT vs. 7% of MM had MRS 0 to 2 at 90 days (RR 2.97, CI 1.60 to 5.57)	mRS shift towards better outcomes at 90 days favoured EVT (OR 2.58, CI 1.60 to 4.15) 17% of EVT vs. 2% of MM had mRS 0 to 2 at 90 days (OR 7.16, CI 2.12 to 24.21)	90-day utility weighted mRS was better in EVT group (2.93 vs. 2.27; OR 0.62, CI −0.09 to 1.34)
Key safety outcomes	Any ICH within 48 h: higher in EVT group (58.0% vs. 31.4%; RR 1.85; CI 1.33 to 2.58) sICH at 48 h higher in EVT group (RR 1.84), no significant difference Mortality at 90 days lower in EVT group, 18% vs. 23.5% (OR 0.77, CI 0.44 to 1.32; *p* 0.33)	Any ICH within 48 h: higher in EVT group (49.1% vs. 17.3%; RR 2.71, CI 1.91 to 3.84) sICH at 48 h higher in EVT group (RR 2.07), no significant difference Mortality at 90 days was 21.7% in EVT vs. 20% in MM (OR 1, CI 0.65 to 1.54)	sICH within 24 h occurred in 1 EVT vs. 2 MM patients (0.6% vs. 1.1%; RR 0.49, CI 0.04 to 5.36) Mortality at 90 days was 38.4% in EVT vs. 41.5% in MM (OR 0.91, CI 0.71 to 1.18)	sICH was 5% in both EVT and MM At least one serious adverse event was 55% in EVT and 70% in MM Death or dependency at 90 days (mRS 4–6) was 69% in EVT vs. 87% in MM (OR 0.34, CI 0.18 to 0.65) Mortality at 90 days was lower in EVT group, 40% vs. 51% (OR 0.67, CI 0.46 to 0.98)	sICH within 24 h was higher in EVT group (3.9% vs. 1.3%; RR 2.96, CI 0.6 to 14.4) Mortality at 90 days similar in both groups (OR 1.06, CI 0.8 to 1.5)

RESCUE-Japan LIMIT: Recovery by Endovascular Salvage for Cerebral Ultra-acute Embolism–Japan Large Ischemic Core Trial; ANGEL-ASPECT: Endovascular Therapy in Acute Anterior Circulation Large Vessel Occlusive Patients with a Large Infarct Core; SELECT2: Randomised Controlled Trial to Optimise Patient’s Selection for Endovascular Treatment in Acute Ischemic Stroke; TENSION: The Efficacy and Safety of Thrombectomy in Stroke with extended lesion and extended time window; TESLA: Thrombectomy for Emergent Salvage of Large Anterior Circulation Ischemic Stroke; EVT: endovascular therapy; MM: medical management; NCCT: non-contrast CT; DWI: diffusion-weighted imaging; ASPECTS: Alberta stroke programme early CT score; CTP: CT perfusion; LKW: last known well; mRS: modified Rankin scale; RR: relative risk; CI: confidence interval; OR: odds ratio; ICH: intracranial haemorrhage; sICH: symptomatic intracranial haemorrhage.

## Data Availability

Not applicable.
